# Vertical farming increases lettuce yield per unit area compared to conventional horizontal hydroponics

**DOI:** 10.1002/fes3.83

**Published:** 2016-06-06

**Authors:** Dionysios Touliatos, Ian C. Dodd, Martin McAinsh

**Affiliations:** ^1^ The Lancaster Environment CentreLancaster UniversityLancasterUK

**Keywords:** Land use efficiency, plant factory, urban agriculture, vertical column grower.

## Abstract

Vertical farming systems (VFS) have been proposed as an engineering solution to increase productivity per unit area of cultivated land by extending crop production into the vertical dimension. To test whether this approach presents a viable alternative to horizontal crop production systems, a VFS (where plants were grown in upright cylindrical columns) was compared against a conventional horizontal hydroponic system (HHS) using lettuce (*Lactuca sativa L*. cv. “Little Gem”) as a model crop. Both systems had similar root zone volume and planting density. Half‐strength Hoagland's solution was applied to plants grown in perlite in an indoor controlled environment room, with metal halide lamps providing artificial lighting. Light distribution (photosynthetic photon flux density, PPFD) and yield (shoot fresh weight) within each system were assessed. Although PPFD and shoot fresh weight decreased significantly in the VFS from top to base, the VFS produced more crop per unit of growing floor area when compared with the HHS. Our results clearly demonstrate that VFS presents an attractive alternative to horizontal hydroponic growth systems and suggest that further increases in yield could be achieved by incorporating artificial lighting in the VFS.

## Introduction

The global population is expected to reach 9 billion by 2050, a significant proportion of which will be urban dwellers, requiring a 70% increase in agricultural productivity (Corvalan et al. 2005; Tilman et al. 2011). Continued rural to urban migration is predicted to drive the expansion of urban landscapes and accelerate the loss of cultivated land surrounding towns and cities (Pandey and Seto 2015). Coupled with land degradation and loss of soil fertility, due to land‐use intensification and climate change, agricultural land is increasingly becoming a scarce resource (Foley et al. 2011; Lambin et al. 2013) in addition to being a threat for biodiversity (Chaplin‐Kramer et al. 2015). In light of the above, the need for innovation in land‐use efficiency for crop production is therefore increasingly important (Lambin and Meyfroidt 2011).

Vertical farming has been proposed as an engineering solution to increase productivity per area by extending plant cultivation into the vertical dimension, thus enhancing land use efficiency for crop production (Eigenbrod and Gruda 2014). The large‐scale implementation of vertical farming involves stacking growth rooms, such as glasshouses and controlled environment rooms, on top of each other to construct food‐producing high‐rise buildings (Despommier 2011). The same concept can be applied at a smaller scale through vertical farming systems (VFS). These growth systems expand crop production into the vertical dimension to produce a higher yield using less floor area (Hochmuth and Hochmuth 2001; Resh 2012). Examples of VFS include the use of vertical columns (Linsley‐Noakes et al. 2006), vertically suspended grow bags (Neocleous et al. 2010), conveyor‐driven stacked growth systems (Mahdavi et al. 2012), A‐frame designs (Hayden 2006), and plant factory approaches (Kato et al. 2010).

Although these studies have quantified crop production, there have been few direct comparisons with horizontal systems of similar cropping density and little information is available on whether vertical column systems present a viable alternative to horizontal crop production systems. In addition, previous yield comparisons of VFS with conventional horizontal systems have confounded other factors with crop orientation. For example, yield increases of 129–200% in VFS and increased profits of 3.6–5.5 US dollar·m^−2^ compared to conventional soil cultivation have been reported (Liu et al. 2004). However, their VFS utilized a soilless growing medium; rendering the comparison essentially invalid. Similarly, significantly higher yields have been reported for strawberry grown in a vertical column VFS compared to conventional grow bags and a multi‐tiered VFS (Ramírez‐Gómez et al. 2012); although no information was provided regarding the root zone volume of the growth systems.

The aim of this study was to compare a vertical column VFS and a conventional horizontal hydroponic system (HHS) with similar fertigation regimes, root zone volumes, and planting densities to determine whether VFS represents a viable alternative to HHS. Lettuce was used as a model plant as it is widely grown in hydroponics as a rapidly growing leafy vegetable (Safaei et al. 2015) thereby avoiding some of the complexities of changes in crop biomass allocation during the reproductive process (Heller et al. 2014). The study was conducted indoors using only artificial lighting, as this is the dominant approach found in most urban vertical farming projects, especially in plant factory designs (Kang et al. 2014; He et al. 2015) and allows more precise control of environmental conditions (Poorter et al. 2012). Our results show that VFS increased lettuce yield per unit area compared to HHS and suggest that variation in light intensity between cropping systems of different spatial orientation could explain differences in crop yield.

## Materials and Methods

### Location

The study was conducted in a 3.4 m × 4.15 m walk‐in Controlled Environment room (CE room) at the Lancaster Environment Centre (LEC, Lancaster University, UK). Illumination was provided by 12 400 W metal halide lamps (HQI‐T 400N; Osram, St Helens, UK) for a 16 hour photoperiod (06.00 h to 22.00 h). Highly reflective plastic film (LBS Horticulture Ltd, Lancashire, UK) was placed on the walls of the room in order to increase the diffusion of light. Room air temperature ranged between 16 and 18 °C and relative humidity ranged from 60% to 80%. Room temperature and humidity were recorded by an Ektron II C sensor (HortiMaX B.V., Pijnacker, the Netherlands), which was hanging from the ceiling in the middle of the CE room, at 1.83 m above the ground. The CE room accommodated 2 VFS and 2 HHS, with one of each arranged on each side of the room. Preliminary measurements of photosynthetic photon flux density (PPFD) (before and after installing the VFS) revealed no shading effect of the VFS on the PPFD in the HHS (data not shown).

### The vertical farming system

Plants were grown vertically in upright cylindrical columns comprised of individual modular units stacked on top of each other to reduce the system footprint. Each modular unit consisted of two stackable elements: a growing container (10.5 cm high and 7.5 cm radius) and a spacing collar (20 cm high and 7.5 cm radius). Five growing containers and six spacing collars were sterilized in TriGene disinfectant (MediChem International Ltd., Sevenoaks, UK) prior to filling each container with 130 g ± 0.5 g medium grade perlite (LBS Horticulture Ltd, Lancashire, UK). Highly porous perlite was the substrate of choice in order to minimize the risk of root‐zone hypoxia and the resultant accumulation of ethylene within the airspace of the vertical column. The perlite was held in place by horticultural frost fleece (LBS Horticulture Ltd, Lancashire, UK) that was placed at the bottom of each growing container. The perlite in each container was levelled and seedlings were placed on the perlite at 90° to the horizontal. Each VFS contained 20 lettuce plants in total. Distance between the top of the VFSs and the light was 80 cm.

### The horizontal hydroponic system

**Figure 1:**
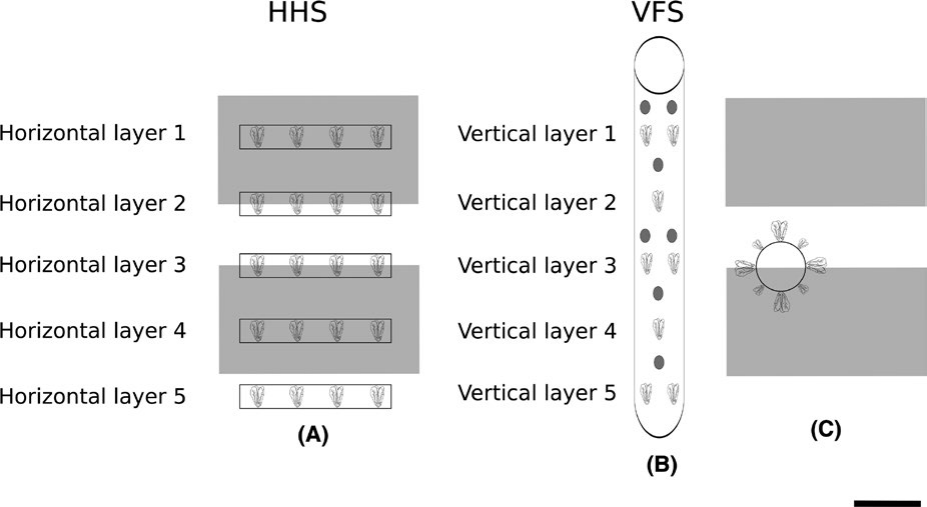
Schematic shows planting densities within the HHS and VFS. (A) Overhead view of HHS. (B) Side‐view of VFS. (C) Overhead view of VFS. The HHS occupied 0.4 m^2^ of growing floor area, whereas the VFS occupied 0.02 m^2^ per column of floor area. The grey rectangles show the exact position of the 400 W metal halide lamps above the growth systems. The grey circles within the VFS show the exact measurement positions of the Macam Q203 Quantum radiometer quantum sensor. Light measurements in the HHS were obtained directly above the plants, at 20 cm distance from the PVC pipes. Scale bar is 20 cm. VFS, vertical farming system; HHS, horizontal hydroponic system.

The HHS comprised of five cylindrical PVC pipes (45.5 cm high and 3.6 cm radius), which were sterilized in TriGene disinfectant (MediChem International Ltd), filled with 130 g ±0.5 g of perlite (LBS Horticulture Ltd, Lancashire, UK) and placed in parallel at 20 cm apart center to center. Horticultural frost fleece (LBS Horticulture Ltd, Lancashire, UK) was placed in the outlet of each pipe to hold the perlite in place. Each pipe held four plants placed in 4.4 cm square holes, in rows. In order to prevent the growth of algae black nylon fabric was used to cover the outlet channel of the system. Each HHS contained 20 lettuce plants in total. PVC pipes were mounted on commercial growth benches (90 cm from the ground and 130 cm from the lights); which was equivalent in height to VFS's layer 2 (Fig. 1).

### Fertigation

Each growth system was supplied with recirculated half‐strength Hoagland's solution (Hoagland and Arnon 1950) from a 18 L Titan PC4R Tank (Kingspan Environmental Ltd, Armagh, UK), through a 1.27 cm double‐walled PVC hose (LBS Horticulture Ltd). The composition of the nutrient solution was 0.5 mmol·L^−1^ NH_4_NO_3_, 1.75 mmol·L^−1^ Ca(NO_3_)_2_·4H_2_O, 2.01 mmol·L^−1^ KNO_3_, 1.01 mmol·L^−1^ KH_2_PO_4_, 0.5 mmol·L^−1^ MgSO_4_·7H_2_O, 1.57 *μ*mol·L^−1^ MnSO_4_·5H_2_O, 11.3 *μ*mol·L^−1^ H_3_BO_3_, 0.3 *μ*mol·L^−1^ CuSO_4_·5H_2_O, 0.032 *μ*mol·L^−1^ (NH_4_)_6_Mo_7_O_24_·4H_2_O, 1.04 *μ*mol·L^−1^ ZnSO_4_·7H_2_O, and 0.25 mmol·L^−1^ NaFe EDTA. Nutrient solution Electrical Conductivity (EC) was 1 ± 0.2 dS·m^−1^. The nutrient solution in tanks was replaced weekly and 2 mol·L^−1^ H_3_PO_4_ was used to maintain a pH of 5.8 ± 0.2, which was checked daily. Hozelock 360° Micro Jet microsprinklers (Hozelock Limited, Aylesbury, UK) delivered the solution to the top layer of the VFS, allowing gravity‐driven drip‐irrigation of plants in growing modules below them in the column. In the HHS the nutrient solution was delivered to each pipe of the HHS by a microsprinkler. The effluent from the bottom layer of the VFS and from all PVC pipes of the HHS was subsequently returned to the tank and recirculated around the growing systems using a submersible aquarium water pump (All Pond Solutions Ltd, Middlesex, UK), capable of delivering a maximum of 3100 L·h^−1^. Hozelock Coupling 13 mm hose connectors (Hozelock Limited, Aylesbury, UK) were used to connect all hoses and pumps. Each pump was programmed to operate for 1 min every hour using a multi purpose electronic digital programmable timer (JoJo Waterproof Digital Outdoor Electrical Timer; AuctionZ Ltd, Bradford, UK). Consequently, the microsprinklers sprayed nutrient solution within the top layers of the VFS and within the horizontal layers of the HHS for 1 min every hour.

### Plant material

Romaine lettuce (*Lactuca sativa* L. cv. ‘Little Gem’) seeds were sown in 84‐cell plug trays (tray dimensions: 53 cm × 31 cm × 5.5 cm) containing Levington M3 compost (Scotts UK, Ipswich, UK). Seedlings were watered daily with tap water and were grown at a PPFD of 200 *μ*mol·m^−2^·s^−1^ over a 16 h photoperiod. Plants were transplanted 20 days after sowing at the four true leaf stage.

### Data collection and statistical analysis

**Figure 2:**
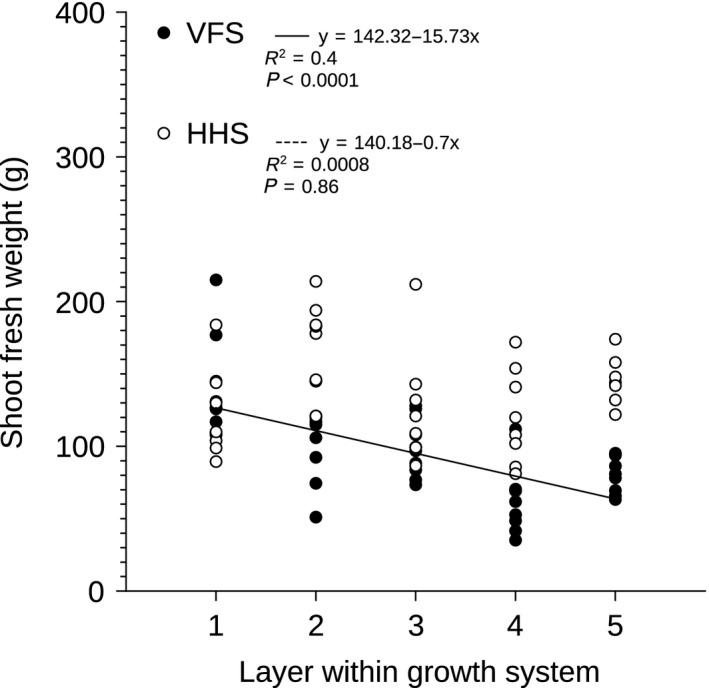
Linear regression analysis of shoot fresh weight versus layer in the VFS (solid line; closed symbols) and HHS (open symbols), respectively. When the linear regression was not significant the regression line was omitted. The regression equation, adjusted *R* ^2^ values and significance of the regression (*P*‐value) are reported at the top of the panel. VFS, vertical farming system; HHS, horizontal hydroponic system.

**Figure 3:**
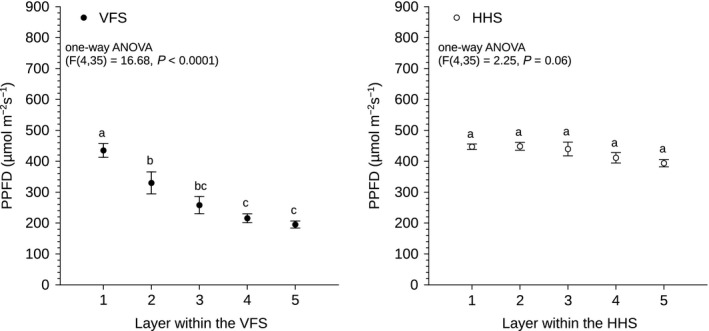
PPFD within the vertical farming system (VFS; closed symbols) and the horizontal hydroponic system (HHS; open symbols) plotted against layers in the growth systems. Values indicated with different letters indicate statistically significant differences, whereas those marked with the same letters show statistically similar values. Error bars represent SE (*n* = 8). VFS, vertical farming system; HHS, horizontal hydroponic system; PPFD, photosynthetic photon flux density.

**Figure 4:**
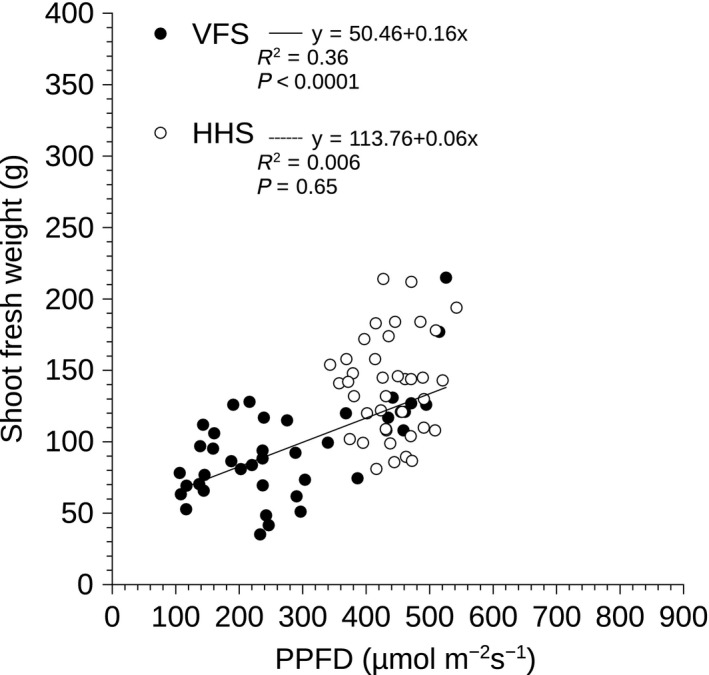
Linear regression analysis of shoot fresh weight versus PPFD in the vertical farming system (VFS; solid line; closed symbols) and horizontal hydroponic system (HHS; open symbols), respectively. When the linear regression was not significant the regression line was omitted. The regression equation, adjusted *R* ^2^ values, and significance of the regression (*P*‐value) are reported at the top of the panel. VFS, vertical farming system; HHS, horizontal hydroponic system; PPFD, photosynthetic photon flux density.

Photosynthetic photon flux density measurements were obtained using a Macam Q203 Quantum radiometer (Macam Photometrics LTD, Livingstone, UK) 1 and 5 weeks after the plants were transferred to the growth systems. The quantum sensor was placed in the middle of the spacing collar, in a 10 cm radius zone around the vertical column in the VFS, and was placed 20 cm above the PVC pipes in the HHS. This approach ensured consistency in light intensity measurements and avoided plant damage during sampling. Shoot fresh weight was measured immediately after harvest on week 5 using a 2 decimal point scientific balance. To compare average shoot fresh weight per growth system using Student's *t*‐test, the data were square root transformed, as they were not normally distributed (Table 1). Yield and number of plants per occupied growing floor area for growth systems was used to calculate the ratio of VFS to HHS (Table 1). Linear regression analysis was used to analyze the relationship between shoot fresh weight and vertical or horizontal layers within each growth system (Fig. 2). Significant differences in PPFD during week 1 within the VFS and the HHS were detected using one‐way ANOVA followed by Tukey post hoc analysis (Fig. 3). The relationship between PPFD during week 5 and shoot fresh weight was analyzed using linear regression analysis (Fig. 4), with *P*‐value <0.05 considered to indicate a statistically significant difference. All statistical tests were performed using “R” version 3.1.2 software (R Development Core Team 2014).

**Table 1:** Comparison of the productivity of the vertical farming system (VFS) and horizontal hydroponic system (HHS)

Parameter	HHS	VFS	Result
Shoot fresh weight (g) Mean ± SE (*n* = 40)	138 ± 6	95 ± 6	*P* < 0.001^b^
Yield per occupied growing floor area^a^ (kg FW·m^−2^)	6.9	95	VFS/HHS = 13.8
Number of plants per occupied growing floor area^a^ (plant number m^−2^)	50	1000	VFS/HHS = 20

a HHS growing floor area: 0.4 m^2^, VFS growing floor area: 0.02 m^2^.

b Student's *t*‐test on square root transformed data, *t* (78) = 5.656.

## Results

### The VFS produced more crop per unit area compared to the HHS

The VFS produced 13.8 times more crop, calculated as a ratio of yield (kg FW) to occupied growing floor area (m^2^). However, mean FW (g) for lettuce crops grown within the HHS was significantly higher than those grown within the VFS. Therefore, although the same number of plants was grown in each system the two HHS produced 1.7 kg more crop compared to the two VFS (5.5 and 3.8 kg of crop in total, respectively). Consequently, the higher productivity of the VFS, in terms of kg FW·m^−2^ growing floor area, can be attributed to the 20‐fold higher number of plants per growing floor area (Table 1).

### Yield decreased from top to base of VFS, whereas yield was uniform within the HHS

Shoot fresh weight decreased from top to base of vertical columns of the VFS, whereas no gradient in productivity was observed between horizontal layers in the HHS. Crop productivity was uniform in the HHS with a range of 133 g within a normal distribution, whereas in the VFS, crop productivity had a range of 180 g within a nonnormal distribution with positive skewness (Sk = 1.035). Plants grown within the top layer of the VFS and within all layers of the HHS were of similar shoot fresh weight. However, in the middle and bottom layers (Layers 2–5) of the VFS, productivity decreased significantly. As a result, the bottom layer of the VFS produced 43% less shoot fresh weight than the top layer of the VFS (Fig. 2).

### Light intensity decreased from top to base of VFS

Light intensity decreased significantly from top to base of vertical columns within the VFS, whereas no significant difference in PPFD was observed within the horizontal layers of the HHS. PPFD values varied between 491 and 134 *μ*mol·m^−2^·s^−1^ from top to base of vertical column of the VFS and between 570 and 340 *μ*mol·m^−2^·s^−1^ within the horizontal layers of the HHS. The top layer of the VFS received similar PPFD to all the horizontal layers of the HHS. However, within the vertical layers, as distance from the light source increased, there was a significant drop in PPFD values within the VFS. There was no significant difference in PPFD between layers 2 and 3 and between layers 3, 4, and 5 (Fig. 3).

### Light intensity influenced growth in the VFS but not in the HHS

There was a significant positive relationship between shoot fresh weight and PPFD in the VFS, indicating that as light intensity increased so did crop productivity (Fig. 4). In contrast, there was no significant relationship between yield and PPFD within the HHS.

## Discussion

Although it has been proposed that increases in yield per growing area can be achieved by extending plant cultivation into the vertical dimension using VFS (Eigenbrod and Gruda 2014), to date there is no conclusive evidence that this is indeed the case. However, our results show that crop productivity, defined as a ratio of yield to occupied growing floor area, is 13.8 times higher in VFS than the HHS. This is likely because by incorporating the vertical dimension into the growth environment, the VFS can grow 20‐fold more plants per unit area than the HHS (Table 1). However, these calculations are based on independent vertical columns and do not consider the effect of column spacing on yield per occupied growing floor area. For example, in high wire crop training systems high planting densities impose intense competition for light within the growth system (Pettersen et al. 2010). This is an important factor that needs to be considered in future studies, as spacing between vertical columns influenced crop productivity in VFS glasshouse trials (Liu et al. 2004).

In contrast, the absolute yield of the HHS, in terms of shoot fresh weight, was higher than the VFS (Table 1). This can be explained by the significant decrease in PPFD from top to base of the vertical columns (Fig. 3) and significant causal relationship between shoot fresh weight and PPFD within the VFS (Fig. 4) that limited growth in the lower layers. Light intensity is one of the primary variables affecting lettuce yield and quality (Ferentinos et al. 2000; Son and Oh 2013; Ouzounis et al. 2015) and it is has been well documented that lettuce yield increases with increasing light intensity (Knight and Mitchell 1988; Frantz and Bugbee 2005). Therefore, since yield decreased from top to base of the vertical column, and yield was uniform within the HHS (Fig. 2), it was anticipated that the VFS would produce less crop in total than the HHS. Similarly, light intensity and shoot fresh weight were highly correlated and both decreased from top to base of vertical columns in a glasshouse (Liu et al. 2004). Light gradients from top to base of vertical column systems were also reported in glasshouse vertical strawberry cultivation (Ramírez‐Gómez et al. 2012). Therefore, our data suggest that top to base gradients in light intensity and shoot fresh weight limit plant growth in vertical columns in both indoor and glasshouse settings.

Vertical light intensity gradients (e.g. Fig. 3) could be altered by natural illumination. In glasshouse trials with vertical columns, light intensity decreased from top to base of vertical columns, with lower PPFD values being recorded toward the northern side of columns compared to the southern side (Liu et al. 2004). Thus, natural illumination introduced an additional gradient in light distribution within the VFS. Future studies are therefore required to test whether natural illumination diminishes or exacerbates light intensity gradients.

Light intensity in growth chambers is known to decrease as distance from the light source increases (Poorter et al. 2012) and this phenomenon partially explained the large variance in PPFD observed in vertical layers 2–5 within the VFS (Fig. 3). In addition, a “shading effect” within the VFS (Linsley‐Noakes et al. 2006) was due to higher positioned plants within the VFS obscuring lower positioned plants from the light source. Side‐on rather than top‐down illumination could potentially ameliorate the shading effect, consequently mitigating the gradient in crop productivity within the VFS. Side‐on illumination, also known as interlighting has improved light distribution within tall canopies and, in some cases, increased crop yield and light use efficiency (Olle and Viršile 2013). Interlighting with light‐emitting diodes (LEDs) ameliorated mutual shading within tomatoes at high planting density and increased tomato yield by 12–14% in comparison to the control (Lu et al. 2012). Overhead illumination combined with intracanopy lighting using HPS lamps increased cucumber yield in high‐wire crop training system by 11% compared to traditional overhead illumination (Pettersen et al. 2010). In contrast, there were no differences in productivity when comparing LED interlighting against overhead HPS in high‐wire tomato cultivation (Gómez et al. 2013). Similarly, interlighting by fluorescent tubes improved fruit quality but did not increase yield in high‐wire cucumber production in the glasshouse (Heuvelink et al. 2006). This variation in crop responses to interlighting may be due to the different environmental conditions and crop management applied. However, since vertical column systems share similar light distribution properties within the vertical plane to plants grown in high wire crop training systems (Hovi et al. 2004), side‐on illumination could potentially mitigate observed light gradients within the VFS.

Interestingly, only 36% of the variation in shoot fresh weight within the VFS was explained by the gradient in PPFD (Fig. 4) with the remaining 64% of variance being attributed to putative temperature gradients and putative nutrient concentration gradients along the vertical column (Jones 2014). Nutrient concentration gradients within the gully of Nutrient Film Technique (NFT) systems have been claimed to influence crop uniformity within the NFT (Puerta et al. 2007). An important difference between the VFS and the HHS of this study was that each layer of the HHS received nutrient solution directly from the tank whereas, in the VFS nutrient solution was delivered to the top layer and was gravity‐driven drip‐fed to vertical layers beneath creating the potential for marked gradients in nutrient availability within the VFS. Identifying the physiological effects of this putative nutrient concentration gradient to growth within VFS is therefore an important area for future studies.

To conclude, from a commercial point of view, the effects of gradients within the VFS on crop value will depend on how the crop is going to be processed and marketed. For example, if lettuce was grown to be sold as individual heads, then the nonuniform productivity of the VFS would be a potential weakness of the VFS over the HHS. However, if the crop was destined for precut salad bags then crop uniformity may be irrelevant while increased yield per unit area could be a significant advantage of the VFS. Therefore, crop utilization and marketability and an investigation of the cost‐to‐benefits ratio of these growing systems will be the ultimate criteria to decide whether VFS can provide an alternative to HHS.

## Conclusions

Vertical column‐based VFS presented a viable alternative to conventional horizontal growth systems by optimizing growing space use efficiency, thereby producing more crop per unit area. Further increases in yield could be achieved by incorporating artificial lighting within the VFS to mitigate the observed PPFD gradient. Future studies could investigate the influence of putative additional gradients (such as root and canopy zone temperature and nutrient concentration gradients) within the VFS and interactions between vertical columns in terms of competition for PPFD in large‐scale vertical farming settings.

## Conflict of Interest

None declared.

## References

[fes383-bib-0001] Chaplin‐Kramer, R. , R. P. Sharp , L. Mandle , S. Sim , J. Johnson , I. Butnar , et al. 2015 Spatial patterns of agricultural expansion determine impacts on biodiversity and carbon storage. Proc. Natl Acad. Sci. USA 112:7402–7407. 26082547 10.1073/pnas.1406485112 PMC4475955

[fes383-bib-0002] Corvalan, C. , S. Hales , and A. J. McMichael . 2005 Ecosystems and human well‐being: health synthesis. World Health Organization.

[fes383-bib-0003] Despommier, D. 2011 The vertical farm: controlled environment agriculture carried out in tall buildings would create greater food safety and security for large urban populations. J. Für Verbraucherschutz Leb. 6:233–236.

[fes383-bib-0004] Eigenbrod, C. , and N. Gruda . 2014 Urban vegetable for food security in cities. A review. Agron. Sustain. Dev. 35:483–498.

[fes383-bib-0005] Ferentinos, K. P. , L. D. Albright , and D. V. Ramani . 2000 SE – structures and environment: optimal light integral and carbon dioxide concentration combinations for lettuce in ventilated greenhouses. J. Agric. Eng. Res. 77:309–315.

[fes383-bib-0006] Foley, J. A. , N. Ramankutty , K. A. Brauman , E. S. Cassidy , J. S. Gerber , M. Johnston , et al. 2011 Solutions for a cultivated planet. Nature 478:337–342. 21993620 10.1038/nature10452

[fes383-bib-0007] Frantz, J. M. , and B. Bugbee . 2005 Acclimation of plant populations to shade: photosynthesis, respiration, and carbon use efficiency. J. Am. Soc. Hortic. Sci. 130:918–927.

[fes383-bib-0008] Gómez, C. , R. C. Morrow , C. M. Bourget , G. D. Massa , and C. A. Mitchell . 2013 Comparison of intracanopy light‐emitting diode towers and overhead high‐pressure sodium lamps for supplemental lighting of greenhouse‐grown tomatoes. HortTechnology 23:93–98.

[fes383-bib-0009] Hayden, A. L. 2006 Aeroponic and hydroponic systems for medicinal herb, rhizome, and root crops. HortScience 41:536–538.

[fes383-bib-0010] He, J. , L. Qin , Y. Liu , and T. W. Choong . 2015 Photosynthetic capacities and productivity of indoor hydroponically grown *Brassica alboglabra* bailey under different light sources. Am. J. Plant Sci. 6:554.

[fes383-bib-0011] Heller, H. , A. Bar‐Tal , S. Assouline , K. Narkis , S. Suryano , A. de la Forge , et al. 2014 The effects of container geometry on water and heat regimes in soilless culture: lettuce as a case study. Irrig. Sci. 33:53–65.

[fes383-bib-0012] Heuvelink, E. , M. J. Bakker , L. Hogendonk , J. Janse , R. Kaarsemaker , and R. Maaswinkel . 2006 Horticultural lighting in the Netherlands: new developments. In V International Symposium on Artificial Lighting in Horticulture. 711:25–34.

[fes383-bib-0013] Hoagland, D. R. , and D. I. Arnon . 1950 The water‐culture method for growing plants without soil. Circ. Calif. Agric. Exp. Stn. 347:2nd edn.

[fes383-bib-0014] Hochmuth, R. , and G. J. Hochmuth . 2001 A decade of change in Florida's greenhouse vegetable industry: 1991–2001 Pp. 280–283 *in* Proc. Fla. State Hort. Soc.

[fes383-bib-0015] Hovi, T. , J. Näkkilä , and R. Tahvonen . 2004 Interlighting improves production of year‐round cucumber. Sci. Hortic. 102:283–294.

[fes383-bib-0016] Jr Jones, , J. B. 2014 Complete guide for growing plants hydroponically. CRC Press.

[fes383-bib-0017] Kang, J. H. , S. KrishnaKumar , S. L. S. Atulba , B. R. Jeong , and S. J. Hwang . 2014 Light intensity and photoperiod influence the growth and development of hydroponically grown leaf lettuce in a closed‐type plant factory system. Hortic. Environ. Biotechnol. 54:501–509.

[fes383-bib-0018] Kato, K. , R. Yoshida , A. Kikuzaki , T. Hirai , H. Kuroda , K. Hiwasa‐Tanase , et al. 2010 Molecular breeding of tomato lines for mass production of miraculin in a plant factory. J. Agric. Food Chem. 58:9505–9510. 20695489 10.1021/jf101874b

[fes383-bib-0019] Knight, S. L. , and C. A. Mitchell . 1988 Effects of CO_2_ and photosynthetic photon flux on yield, gas exchange and growth rate of *Lactuca sativa* L. “Waldmann”s Green’. J. Exp. Bot. 39:317–328. 11539044 10.1093/jxb/39.3.317

[fes383-bib-0020] Lambin, E. F. , and P. Meyfroidt . 2011 Global land use change, economic globalization, and the looming land scarcity. Proc. Natl Acad. Sci. USA 108:3465–3472. 21321211 10.1073/pnas.1100480108 PMC3048112

[fes383-bib-0021] Lambin, E. F. , H. K. Gibbs , L. Ferreira , R. Grau , P. Mayaux , P. Meyfroidt , et al. 2013 Estimating the world's potentially available cropland using a bottom‐up approach. Glob. Environ. Change 23:892–901.

[fes383-bib-0022] Linsley‐Noakes, G. , L. Wilken , and S. de Villiers . 2006 High density, vertical hydroponics growing system for strawberries. Acta Hortic. 708:365.

[fes383-bib-0023] Liu, W. , D. K. Chen , and Z. X. Liu . 2004 High efficiency column culture system in China. Acta Hortic. 691:495–500.

[fes383-bib-0024] Lu, N. , T. Maruo , M. Johkan , M. Hohjo , S. Tsukagoshi , Y. Ito , et al. 2012 Effects of Supplemental Lighting within the Canopy at Different Developing Stages on Tomato Yield and Quality of Single‐Truss Tomato Plants Grown at High Density. Environ. Control. Biol. 50:1–11.

[fes383-bib-0025] Mahdavi, S. , M. Kafi , R. Naderi , and T. Sadat . 2012 Vertical mobile planting system consistent with the pattern of solar radiation and effects of system on light exposure and growth of Gerbera cut flowers (*Gerbera jamesonii* cv. Antibes), in greenhouse culture. J. Agric. Technol. 8:1461–1468.

[fes383-bib-0026] Neocleous, D. , C. Kaittanis , N. Seraphides , and P. Polycarpou . 2010 Horizontal and vertical soilless growing systems under Cyprus conditions. J. Appl. Hortic. 12:140–144.

[fes383-bib-0027] Olle, M. , and A. Viršile . 2013 The effects of light‐emitting diode lighting on greenhouse plant growth and quality. Agric. Food Sci. 22:223–234.

[fes383-bib-0028] Ouzounis, T. , B. R. Parjikolaei , X. Fretté , E. Rosenqvist , and C. O. Ottosen . 2015 Predawn and high intensity application of supplemental blue light decreases the quantum yield of PSII and enhances the amount of phenolic acids, flavonoids, and pigments in *Lactuca sativa* . Front. Plant Sci. 6. 10.3389/fpls.2015.00019 PMC4341431 25767473

[fes383-bib-0029] Pandey, B. , and K. C. Seto . 2015 Urbanization and agricultural land loss in India: comparing satellite estimates with census data. J. Environ. Manage. 148:53–66, Land Cover/Land Use Change (LC/LUC) and Environmental Impacts in South Asia. 24958549 10.1016/j.jenvman.2014.05.014

[fes383-bib-0030] Pettersen, R. I. , S. Torre , and H. R. Gislerød . 2010 Effects of intracanopy lighting on photosynthetic characteristics in cucumber. Sci. Hortic. 125:77–81.

[fes383-bib-0031] Poorter, H. , F. Fiorani , M. Stitt , U. Schurr , A. Finck , Y. Gibon , et al. 2012 The art of growing plants for experimental purposes: a practical guide for the plant biologist. Funct. Plant Biol. 39:821–838. 10.1071/FP12028 32480833

[fes383-bib-0032] Puerta, A. R. , S. Sato , Y. Shinohara , and T. Maruo . 2007 A modified nutrient film technique system offers a more uniform nutrient supply to plants. Horttechnology 17:227–233.

[fes383-bib-0050] R Core Team 2014 R: A language and environment for statistical computing. R Foundation for Statistical Computing, Vienna, Austria Available at http://www.R-project.org/.

[fes383-bib-0033] Ramírez‐Gómez, H. , M. Sandoval‐Villa , A. Carrillo‐Salazar , and A. Muratalla‐Lúa . 2012 Comparison of hydroponic systems in the strawberry production. Acta Hortic. 947:165–172.

[fes383-bib-0034] Resh, H. M. 2012 Hydroponic food production: a definitive guidebook for the advanced home gardener and the commercial hydroponic grower. CRC Press.

[fes383-bib-0035] Safaei, M. , J. Panahandeh , S. J. Tabatabaei , and A. R. M. Azar . 2015 Effects of different nutrients solutions on nutrients concentration and some qualitative traits of lettuce in hydroponics system. J. Sci. Technol. Greenh. Cult. 6:Pe1–Pe7, En8.

[fes383-bib-0036] Son, K.‐H. , and M.‐M. Oh . 2013 Leaf shape, growth, and antioxidant phenolic compounds of two lettuce cultivars grown under various combinations of blue and red light‐emitting diodes. HortScience 48:988–995.

[fes383-bib-0037] Tilman, D. , C. Balzer , J. Hill , and B. L. Befort . 2011 Global food demand and the sustainable intensification of agriculture. Proc. Natl Acad. Sci. USA 108:20260–20264. 22106295 10.1073/pnas.1116437108 PMC3250154

